# Suppression of Electrographic Seizures Is Associated with Amelioration of QTc Interval Prolongation in Patients with Traumatic Brain Injury

**DOI:** 10.3390/jcm10225374

**Published:** 2021-11-18

**Authors:** Wojciech Dabrowski, Dorota Siwicka-Gieroba, Todd T. Schlegel, Chiara Robba, Sami Zaid, Magdalena Bielacz, Andrzej Jaroszyński, Rafael Badenes

**Affiliations:** 1 Department of Anaesthesiology and Intensive Therapy, Medical University of Lublin, 20-954 Lublin, Poland; dsiw@wp.pl; 2 Department of Molecular Medicine and Surgery, Karolinska Institute, SE-171 76 Stockholm, Sweden; ttschlegel@gmail.com; 3 Nicollier-Schlegel SARL, 1270 Trélex, Switzerland; 4 Department of Anaesthesia and Intensive Care, Policlinico San Martino, 16100 Genova, Italy; kiarobba@gmail.com; 5 Department of Anaesthesia, Al-Emadi-Hospital, Al HilalWest, D Ring Road, Doha P.O. Box 50000, Qatar; sami1zaid@gmail.com; 6 Institute of Tourism and Recreation, State Vocational College of Szymon Szymonowicz, 22-400 Zamosc, Poland; magda.bielacz@gmail.com; 7 Department of Nephrology, Institute of Medical Science, Jan Kochanowski University of Kielce, 25-736 Kielce, Poland; jaroszynskiaj@interia.pl; 8 Department of Anaesthesiology and Intensive Care, Hospital Clìnico Universitario de Valencia, University of Valencia, 46010 Valencia, Spain; rafaelbadenes@gmail.com

**Keywords:** seizure, traumatic brain injury, QTc interval, spatial QTS-T angle, brain–heart interaction

## Abstract

Introduction: Disorders in electroencephalography (EEG) are commonly noted in patients with traumatic brain injury (TBI) and may be associated with electrocardiographic disturbances. Electrographic seizures (ESz) are the most common features in these patients. This study aimed to explore the relationship between ESz and possible changes in QTc interval and spatial QRS-T angle both during ESz and after ESz resolution. Methods: Adult patients with TBI were studied. Surface 12-lead ECGs were recorded using a Cardiax device during ESz events and 15 min after their effective suppression using barbiturate infusion. The ESz events were diagnosed using Masimo Root or bispectral index (BIS) devices. Results: Of the 348 patients considered for possible inclusion, ESz were noted in 72, with ECG being recorded in 21. Prolonged QTc was noted during ESz but significantly ameliorated after ESz suppression (540.19 ± 60.68 ms vs. 478.67 ± 38.52 ms, *p* < 0.001). The spatial QRS-T angle was comparable during ESz and after treatment. Regional cerebral oximetry increased following ESz suppression (from 58.4% ± 6.2 to 60.5% ± 4.2 (*p* < 0.01) and from 58.2% ± 7.2 to 60.8% ± 4.8 (*p* < 0.05) in the left and right hemispheres, respectively). Conclusion: QTc interval prolongation occurs during ESz events in TBI patients but both it and regional cerebral oximetry are improved after suppression of seizures.

## 1. Introduction

Electrocardiographic disorders are frequently associated with seizures, which are often observed in patients with traumatic brain injury (TBI). Post-traumatic seizures occur in 21–27% of patients and are generally associated with hemorrhagic lesions of the temporal lobe [1,2,3]. The first event of recorded seizures mostly occurs in the first 24 h after TBI, with over one-third being electrographic seizures (ESz—electrographic seizures) [1]. TBI-related seizures can also be induced by increased intracranial pressure (ICP), cerebral metabolic crises connected with disturbances in oxidative metabolism and glucose consumption, and impaired redox status of the brain [3]. On the other hand, seizures impair cerebral metabolism and may induce systemic disorders in the extra-cerebral organs, including cardiac injury [4].

Cerebral-related cardiac disorders commonly result from activation of the brain–heart axis, with electrocardiographic disorders being frequent derangements observed in patients with acute brain damage [5,6,7,8]. Recently, significant prolongation in the QTc interval and increased spatial QRS-T angles in a cohort of TBI patients with a Glasgow Coma Score (GCS) below eight were documented [6]. Some studies reported a strict relationship between prolonged QTc interval and seizures based on standard full electroencephalography (EEG) measurement [7,8]. However, no study so far has explored the relationship between TBI-related ESz and changes in the QTc interval or spatial QRS-T angle. The aim of this study was to analyze changes in the spatial QRS-T angle and QTc interval during ESz and after ESz resolution by EEG in a cohort of TBI patients.

## 2. Methods

We used Strengthening the Reporting of Observational Studies in Epidemiology (STROBE) guidelines [9]. This study is part of a larger prospective observational study performed at the First Clinic of Intensive Care at the Medical University of Lublin, Poland. The study was conducted in accordance with the intensive care unit (ICU) protocol for the monitoring of patients with TBI and the Declaration of Helsinki; the protocol was approved by the Institutional Review Board and the Bioethics Committee of the Medical University at Lublin, Poland (KE-0254/136/2018). Informed consent was obtained from the legal representatives of patients as all included patients were sedated and mechanically ventilated.

Inclusion criteria were adult patients with TBI and GCS below 8 and the presence of ESz. The main exclusion criterion was a history of epilepsy. Additionally, patients below 18 years old, pregnant, or with the presence of thoracic injury, drug overdoses, or a history of neoplastic, cardiac, or acute or chronic hepatic or renal diseases were excluded. Heart rate (HR), continuous mean arterial pressure (MAP), regional cerebral oximetry (SrO_2_), and peripheral saturation (SpO_2_) were monitored in all patients; hemodynamic variables were obtained using an EV 1000 platform (Edwards Lifescience, Irvine, CA, USA). Immediately after admission to the ICU, EEG electrodes were placed on the forehead and temporal hairline skin for EEG monitoring using Masimo Root with a SEDLine monitor (Irvine, CA, USA) or a bispectral complete 4-channel monitor (BIS, Medtronic, MN, USA).

All patients were sedated with propofol (AstraZeneca, Macclesfield, UK) and fentanyl (Polfa, Warsaw, Poland) and mechanically ventilated; the inspired fraction of oxygen (FiO_2_) was adjusted to maintain oxygen saturation (SpO_2_) between 92 and 98% and SrO_2_ higher than 50%. Immediately after admission to the ICU, patients received hyperosmotic therapy with 15% mannitol at 1.5 g/kg body weight to reduce ICP, if required. The hyperosmotic therapy was discontinued in patients with plasma osmolality higher than 310 mOsm/kg H_2_O. All patients received continuous infusion of potassium to maintain blood concentration between 4.5 and 5 mmol/L. Blood potassium, sodium, glucose, and lactate levels were measured 5 times per day. Continuous norepinephrine infusion and balanced crystalloids (Sterofundin ISO, Melsungen, Germany) were used to maintain MAP above 80 mmHg. According to the Fourth Edition of Brain Trauma Foundation Guidelines, the infusion of barbiturates is considered an option for second-tier therapy to control refractory elevated intracranial hypertension (ICH) [10].

### 2.1. ECG, Derived Vectorcardiogram (VCG), EEG, and Study Protocol

Surface 12-lead ECGs were recorded using a Cardiax PC-ECG^®^ (MESA Medizintechnic GmbH Benediktbeuern, Germany). The recorded ECG was converted to a single median beat and transformed into three orthogonal leads—X, Y, and Z—using the inverse Dower method [11]. The value for the spatial QRS-T angle was then automatically calculated by the Cardiax software from the maximum spatial QRS and T vectors. The QT and corrected QT (QTc) intervals utilizing the Bazett, Fridericia, and Framingham corrections were also obtained directly from the Cardiax commercial software, which utilizes a median beat-related “global QT interval” algorithm similar to that described by Xue et al. [12]. The QT and QTc intervals were also assessed manually via electronic calipers by two of the co-authors independently to further validate the automatically assessed values. The ECG and derived VCG measurements were performed during ESz events and 15 min after effective suppression of seizures with barbiturate infusion (thiopental, Rotexmadica, Trittau, G) at the dose of 50 µg·kg^−1^·min^−1^.

EEG disorders were analyzed based on frontopolar (forehead hairline montage) EEG recorded with a Masimo Root monitor or a bispectral complete 4-channel (BIS-4) monitor. Both of these technologies are commonly used at our institution to measure the level of sedation. The capacity of the Masimo device for EEG recording has been previously established [13,14,15]. In all patients, changes in EEG were observed within the first 7 days of treatment. Electrographic seizures were defined as electrographic discharges with a frequency higher than 2.5 Hz and lasting longer than 10 s. Seizure morphology was categorized as epileptiform if it induced spikes or sharp waves or rhythmic evolving or if it induced evolving rhythmic patterns [15,16,17]. Electrographic status epilepticus was defined as an ESz for more than 10 continuous minutes or for a total duration of more than 20 min in any 60-minute period of recording [16]. These criteria were adapted for disorders in EEG observed in the BIS or Masimo device. Additionally, changes in EEG were recorded as the color density spectral array (DSA), with upward arches on the y-axis (increased frequency and amplitude in EEG) reflected in warmer colors (larger red area in DSA) [18,19,20].

### 2.2. Statistical Analysis

The Shapiro–Wilk test was used to test the normality of the distribution of the results. Means and standard deviations (SDs) were calculated for all variables. Student’s unpaired *t*-test was used, and analysis was performed using Statistica 13.1 (StatSoft, Tulsa, OK, USA). For variables with non-normal distribution, the Wilcoxon signed-rank, Mann–Whitney U, Kruskal–Wallis ANOVA, and post hoc Dunnett’s multiple comparison tests were used. The power of the statistical tests was assessed by the G*Power test. A *p*-value < 0.05 was considered significant.

## 3. Results

In total, 348 patients (134 female and 214 male) aged 18–90 years and treated for TBI with ICH were initially considered for inclusion. Among these, 115 patients were excluded as they presented with thoracic injury, a history of severe cardiac diseases (N = 111), or pacemaker implantation (N = 4). Finally, 233 patients treated for isolated TBI (iTBI, N = 124) and polytrauma with TBI (pTBI, N = 109) were included to the present study. Eighty-two patients (35.19%) died at day 28, following foraminal herniation (N = 54; 65.85%) or post-traumatic multiorgan failure (N = 28; 33.14%).

Immediately after admission into the ICU, all patients achieved an appropriate level of sedation with continuous propofol and fentanyl infusion, and the depth of sedation ranged between 10 and 20 on the bispectral index (BIS) and 5 and 17 on the Patient State Index (PSI). Any EEG abnormalities were monitored in all patients immediately after admission to the ICU. Episodes of ESz were noted in 72 patients (30.9%) between 12 h and the fifth day after admission in the ICU. In 54 cases, the episodes of ESz were documented on the screenshots in Masimo or BIS-4, and subsequently, ESz was successfully treated; however, changes in ECG during ESz were documented in only 21 patients (7 female and 14 male) aged 19–58 years (mean 39 ± 13). The mean GCS at hospital admission was 4.86 ± 1.6. In all cases, ESz status lasted for more than 15 min before treatment. Of the 21 patients included in the analysis, 11 were treated for cerebral edema (with or without intracerebral hemorrhage), 8 for subarachnoid hemorrhage, and 2 for epidural hematoma. Continuous infusion of thiopental at the dose of 50 µg·kg^−1^·min^−1^ successfully suppressed ESz in all patients, and any EEG abnormalities were observed during and immediately after thiopental administration with a slight decrease in BIS or PSI values. This treatment was continued for a minimum of 12 h. Nine patients died within 28 days of treatment—three due to foraminal herniation within 7 days of treatment and six after 7 but before 28 days due to foraminal herniation. Twelve patients were discharged from the ICU; however, all remained bedridden with neurological conditions.

**Figure 1:**
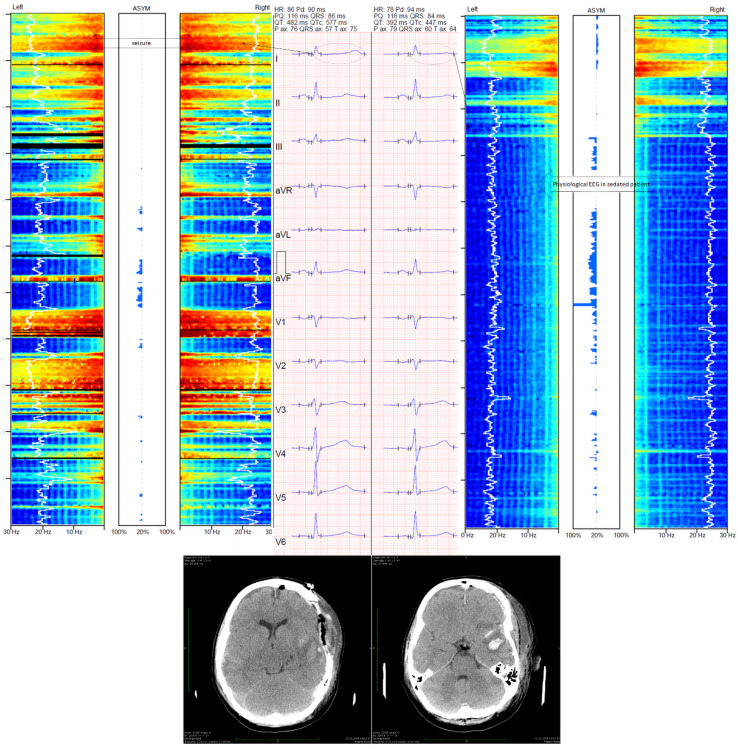
Changes in frontopolar electroencephalography monitored with Medtronic BIS ™ device and corrected QT (QTc) interval. The left part of the figure presents an electrographic seizure and ECG with prolonged QTc (577 ms, calculated with Bazett’s formula). The right part of the figure presents ESz suppression following thiopental administration with reduction in the QTc interval (447 ms, calculated with Bazett’s formula). The case shown is a 22 year-old woman who was admitted to the intensive care unit (ICU) for severe TBI. Her Glasgow Coma Score was 6. Computed tomography (CT) showed acute epidural hematoma with intracerebral hemorrhage. Immediately after CT, craniectomy was performed. According to the local ICU protocol, frontopolar electroencephalography (EEG) was used. Controlled CT was performed 24 h after surgery and showed slight cerebral edema with cerebral lesion and intracerebral hematoma in the temporal region. Despite depth sedation (BIS ranged between 10 and 20), EEG showed alternate polyspike and slow wave without clinical symptoms 24 h after the admission to the ICU. The ESz recurred for 2 h. Hence, status epilepticus was diagnosed and continuous thiopental infusion at the dose of 50 µg·kg^−1^·min^−1^ was used to suppress ESz. Such disorders were not observed during treatment in the ICU. Patient was discharged from the ICU 32 days after trauma.

**Figure 2:**
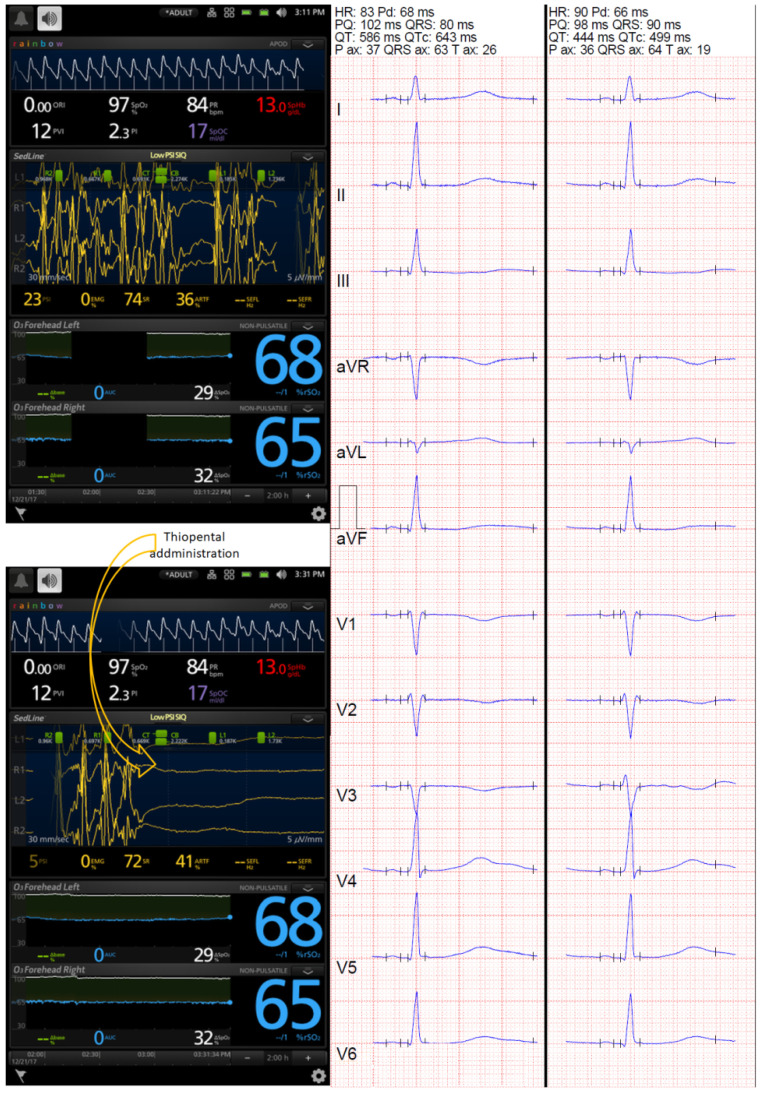
Changes in frontopolar electroencephalography monitored with Masimo Root device and corrected QT (QTc) interval. Prolonged QTc interval was noted during ESz, and use of thiopental suppressed the seizure successfully, which was associated with a reduction in the automated QTc (from 643 to 499 ms, calculated with Bazett’s formula, and from 610 to 475 ms and 591 to 471 ms, calculated with Fridericia’s formula and Framingham’s formula, respectively). However, the ECG showed bifid T waves in V_4_, V_5_, and V_6_ leads before suppression and in II, III, aVF, V_3_, V_4_, V_5_, and V_6_ 15 min after ESz suppression, leading to partially spurious automated QTc results. Frontopolar EEG monitoring with the Masimo device showed seizures (upper screenshot) and their spectacular suppression following barbiturate infusion (lower screenshots).

**Figure 3:**
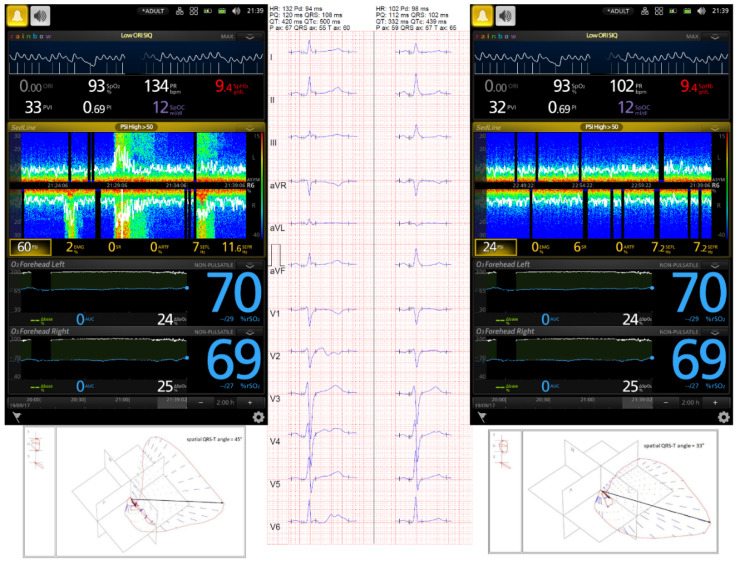
A 54-year-old male admitted to the intensive care unit (ICU) for severe TBI. His Glasgow Coma Score was 4. Computed tomography (CT) showed cerebral edema with reduced size of both lateral ventricles. Patient was sedated with continuous infusion of propofol and fentanyl, and hyperosmotic therapy with 15% mannitol was administered. According to the local ICU protocol, non-invasive monitoring including near-infrared spectroscopy (NIRS) and frontopolar electroencephalography (EEG) was applied. Frontopolar EEG and seizures were monitored by the Masimo Root SEDLine device. Despite deep sedation (Patient State Index was 7), the DSA image showed upward y-axis arcs in warmer colors, and the recorded EEG confirmed a polyspike and slow wave. Continuous thiopental infusion at the dose of 50 µg·kg^−1^·min^−1^ was used to suppress ESz, after which a serial 12-lead ECG showed a notably reduced QTc interval. The patient was discharged from the ICU 14 days after trauma.

During ESz, the QTc interval was pathologically prolonged, and the prolongation was significantly ameliorated 15 min after ESz suppression independently of the method (automated or manual) or correction formula used in all patients (Table 1). Examples of changes in EEG monitored by BIS-4 are presented in Figure 1, and the ESz and ECG monitored by Masimo Root are presented in Figure 2 and Figure 3. Spatial QRS-T angle was comparable during ESz and 15 min after ESz suppression (61.29 ± 46.51 and 60.41 ± 39.73, respectively).

The mean values of cardiac index (CI), extravascular lung water index (ELWI), pulmonary vascular permeability index (PVPI), global ejection fraction (GEF), and intrathoracic blood volume were comparable before and after ESz suppression, whereas the mean values of SrO_2_ significantly increased 15 min after treatment of ESz (Table 2).

## 4. Discussion

The present study showed that observed electrographic abnormalities which may meet the criteria for diagnosis of ESz were associated with T wave abnormalities and prolonged QTc interval in propofol-sedated TBI patients, and that the successful treatment of ESz ameliorated the T wave abnormalities and shortened the QTc interval. Neither ESz nor successful ESz treatment appeared to modify the spatial QRS-T angle. Additionally, treatment of ESz improved SrO_2_ in the left and right hemispheres. Secondarily, this study also demonstrated the potential usefulness of the Masimo and BIS technology for innovative cerebral monitoring in critically ill patients with TBI. Despite all the patients being deeply sedated, the Masimo device enabled identification of general electrographic abnormalities.

TBI-related cerebral hypoxia and ischemia can disrupt autonomic self-regulation and serve as a trigger for seizure development [21]. A rapid change in EEG oscillation may also correspond with changes in cardiac activity and different types of arrhythmias [22,23]. Seizures might affect cardiac function by impairing the repolarization phase, particularly in patients treated for refractory epilepsy [24,25]. Various types of cardiac arrhythmias have been observed in more than 90% of patients with seizures, with atrial fibrillation (AF) being the most frequent form of dysrhythmia [25]. Interestingly, the administration of anti-convulsive drugs significantly reduces ECG disorders and improves cardiac morphology in experimental models of seizures [26]. Seizure-related ventricular arrhythmias are also commonly associated with prolonged QTc interval, possibly due to hypoxemia following seizure-related respiratory dysfunction [8]. Indeed, intermittent hypoxia has been shown to be a major risk factor for life-threatening cardiac arrhythmias and sudden cardiac death [7]. Our results did not explore hypoxia-related QTc interval prolongation as the patients included in our study were mechanically ventilated and cerebral oxygenation was monitored using SrO_2_, without showing any episodes of desaturation. However, significantly lower SrO_2_ levels were noted during ESz compared to post-seizure. Based on these results, we speculate that suppression of electrographic seizures can improve cerebral oxygenation, but this effect requires further confirmation in larger studies.

Continuous monitoring of electrophysiological function in critically ill patients has been extensively studied in the last decade [15,18,19]. In the present study we utilized a new and convenient method for assessing cerebral EEG in critically ill patients treated for TBI. Continuous monitoring of EEG, particularly with DSA that is displayed on processed EEG, shows the power spectrum of EEG, which may be useful for detecting even short episodes of ESz. Detection of ESz in deeply sedated patients is difficult and requires continuous EEG monitoring. To facilitate the interpretation of EEG, several quantitative EEG display tools have been developed to diagnose EEG disorders, and DSA is one of them [15,20,27]. It has been documented that even non-physician personnel can identify ESz in DSA [18,19,27]. Additionally, such qualitative techniques show a high sensitivity, specificity, and accuracy for seizure detection in those personnel who have no experience in critical care EEG and seizure detection [18,19]. Compared to the gold standard of raw data read by experienced epileptologists, Dericioglu and colleagues documented an overall sensitivity of 93%, specificity of 91–95%, and accuracy of 0.93 of DSA in the detection of ESz [18]. Similarly high sensitivity and specificity in the detection of seizures using color DSA were described by Steward and colleagues [28]. Seizure evolution may be accompanied by increases in the frequency and amplitude of frontotemporal EEG signals that appear in DSA images, showing upward y-axis arcs in warmer colors [20,26]. In our cases, we observed that the color spectrum ranged from blue to dark red (from minimum to maximum power), which could document seizure events [15]. Continuous monitoring of EEG waves also enabled the detection of polyspike waves associated with ESz in deeply sedated patients [15,18]. We noted ESz in 30.9% of patients; however, we were able to document changes in ECG during ESz only in one-third of the studied patients because the seizure resolved spontaneously or the doctor on duty documented the seizure episode in the screenshot and implemented continuous thiopental infusion before ECG examination. Based on our experience, we can strongly recommend the use of continuous EEG monitoring in critically ill, unconscious TBI patients.

Other potential mechanisms underlying seizure-induced QTc interval prolongation include the stimulation of the intrinsic adrenergic pathway, leading to disorders in cardiac repolarization, also previously described in ESz [29]. Autonomic dysregulation following TBI might also contribute to QTc interval prolongation [5]. Finally, moderate–severe TBI with hemorrhagic contusions in the temporal lobe increases the risk of early seizure and post-traumatic epilepsy [2]. In the present study, we did not analyze the relationships between episodes of ESz and regions of brain injury; however, we can speculate that a prolonged QTc interval is directly associated with abnormal electroencephalographic brain activity, which could suggest brain–heart interaction [30].

Several medications may also prolong the QTc interval [31,32]. The included patients were sedated using continuous propofol and fentanyl infusion, and both drugs may affect the QTc interval. Propofol is well known to increase the risk of Torsades de Pointes dysrhythmia, which can frequently induce sudden cardiac death [33,34]. Additionally, propofol may cause prolongation of the QTc interval and result in a higher incidence of bradycardia and junctional rhythm than barbiturates can [31]. In the present study, some patients received furosemide to force diuresis, a loop diuretic that could also prolong the QTc interval [29,32]. Notably, furosemide and fentanyl only rarely prolong the QTc interval in critically ill patients, especially in association with electrolyte disturbances [33]. Interestingly, we noted a significant reduction in the QTc interval after ESz suppression with thiopental, a drug known to induce QTc interval prolongation [32]. Therefore, we can speculate that changes in QTc interval may depend more on the seizures induced by the primary cerebral pathology than on the medications administered.

### Limitations

The first major limitation of the present study is that EEG was limited to the frontopolar region because both the Masimo and BIS-4 monitors only allow EEG monitoring in the frontal and temporal lobes. It should also be stressed that EEG abnormalities occurring in the other regions of the brain may also affect ECG as well as stimulate the frontal and temporal lobes to pathological activities. Additionally, changes in the color spectrum may reflect changes in EEG and ICP-related changes but also rhythmic/periodic artefacts, thus reducing the accuracy of quantitative EEG measurement. The second major limitation was that the EEG signal was evaluated by physicians without formal training in neurophysiology but with a lot of experience in the use of quantitative EEG and DSA. Thirdly, all EEG abnormalities were observed in the Masimo or the BIS device and were not confirmed in a standard EEG, but only full EEG could confirm a diagnosis of every electrographic pathology. Another important limitation of the study is the low power of our statistical analysis due to the small number of patients who had both ECG recordings and well-documented ESz. The small number of patients with well-documented ECG during ESz results in part from the lack of specific alarms for EEG disorders in the Masimo technology. Had such alarms existed, they might have allowed for more prompt recognition and treatment of cerebral electrical derangements as well as a larger study group. We did not analyze the duration of ESz in relation to the ECG, and it has been documented that long-standing seizures may induce cardiac remodeling with altered intracellular Ca^+2^ homeostasis and ECG abnormalities, including QTc interval prolongation [35]. We monitored frontopolar EEG for 7 days of treatment. Although we did not observe recurrent episodes of ESz during the study period, they could have recurred after the studied period. Finally, we noted a significant improvement of SrO_2_ following ESz suppression; however, the increase in SrO_2_ in the left and right hemispheres may also have resulted from a decrease in ICP following thiopental administration. In fact, a moderate inverse relationship between ICP and SrO_2_ has been documented in TBI patients [36,37]. Another important limitation of our study is the lack of a control group. We used continuous thiopental infusion to ameliorate ESz; however, many other medications are also commonly used for the treatment of such disorders (e.g., valproic acid or benzodiazepines). Noteworthily, all of these drugs may affect QTc. Therefore, changes in QTc should be compared with those observed in patients with spontaneous ESz termination, because only this analysis would unambiguously confirm the relationships between brain pathology and cardiac dysfunction, which is known as brain–heart interaction.

Our report focused in particular on automated measurements for the QT and QTc intervals. However, no automated algorithm for QTc determination produces perfect results, especially in the face of poorly defined T waves. While our manual measurements of the QTc intervals confirmed the overall QTc-related changes, they did so with less statistical significance, suggesting that methodological (i.e., automated algorithmic) factors can also falsely contribute to increases in automated QTc intervals when T waves are or become very poorly defined. Additionally, we observed significant shortening of the QTc interval following pharmacological suppression of ESz; however, we did not perform ECGs before the seizures, and prolonged QTc intervals might also result from TBI. Nonetheless, the study corroborated that the brain–heart interaction and QTc interval prolongations previously noted during/after seizure activity unrelated to TBI can also occur after TBI. Specifically, it demonstrated not only a strong relationship between TBI-induced ESz and prolonged QTc intervals but also the amelioration of prolonged QTc intervals after the treatment of ESz even in the face of QTc interval-prolonging barbiturate (thiopental) therapy in sedated TBI patients. Our findings support the concept of brain–heart cross-talk [38,39,40,41,42,43,44,45] and may pave the way to further larger observational or randomized controlled studies on this topic.

## 5. Conclusions

Pharmacological suppression of electrographic abnormalities which may meet the criteria to detect ESz in patients with severe TBI is associated with electrocardiographic T wave changes and shortened QTc intervals noted 15 min after seizure suppression. Additionally, treatment of electrographic seizures appears to improve SrO_2_ in both the left and right cerebral hemispheres. Our findings may support that pathological brain activity affects cardiac function, which is commonly known as brain–heart cross-talk. Additionally, we suggest that continuous monitoring of EEG can be useful to detect electrographic abnormalities. However, the relationship between electrographic abnormalities and ECG requires further study in TBI patients.

## Figures and Tables

**Table 1:** Changes in QT and QTc intervals during electrographic seizures and 15 min after their effective suppression with barbiturate infusion. * *p* < 0.05, ** *p* < 0.01, *** *p* < 0.001—difference between QT and QTc before and after suppression of seizure (Student’s *t*-test). Manual measurements comprise the averaged values from two independent co-authors.

	Manual Measurements		Automatic Measurements	
	During ESz	After ESz	During ESz	After ESz
**QT**	453.5 ± 66.3	416.3 ± 55.44	450.71 ± 68.9	410.14 ± 53.95 *
**QTc, Bazett**	544.24 ± 57.67	487.61 ± 40.37 ***	540.19 ± 60.68	478.67 ± 38.52 ***
**QTc, Fridericia**	511.71 ± 55.96	462.16 ± 41.95 **	507.9 ± 59	455.2 ± 39.8 **
**QTc, Framingham**	499.67 ± 51.91	457.19 ± 40.47 *	496.54 ± 54.6	451 ± 38.7 **

**Table 2:** Changes in cardiac index (CI), extravascular lung water index (ELWI), pulmonary vascular permeability index (PVPI), global ejection fraction (GEF), intrathoracic blood volume (iTBI), and regional cerebral oxygenation (SrO_2_) in the right and left hemispheres during ESz and 15 min after their suppression with continuous thiopental infusion at the dose of 50 µg·kg^−1^·min^−1^. * *p* < 0.05, ** *p* < 0.01—differences in SrO_2_ noted before and after suppression of ESz.

Parameter	During ESz	15 min after Thiopental Administration
CI (L/min/m^2^)	3.4 ± 0.8	3.7 ± 0.9
ELWI (mL/kg)	8.2 ± 1.8	7.6 ± 2
PVPI	2.1 ± 0.5	1.9 ± 0.5
GEF (%)	27.2 ± 6.8	28.9 ± 6.4
iTBI (mL/m^2^)	781.4 ± 250.4	804.4 ± 236.2
Left SrO_2_ (%)	58.4 ± 6.2	60.5 ± 4.2 **
Right SrO_2_ (%)	58.2 ± 7.2	60.8 ± 4.8 *

## Data Availability

The data presented in this study are openly available with the author.
